# NUTS2 level dataset on land use and land cover projections for Europe under the shared socio-economic pathways through 2050

**DOI:** 10.1016/j.dib.2025.112268

**Published:** 2025-11-17

**Authors:** Theresa Goebel, Wolfgang Britz

**Affiliations:** Institute for Food and Resource Economics, University of Bonn, Nussallee 21, 53115 Bonn, Germany

**Keywords:** Computable general equilibrium (CGE) modelling, Recursive-dynamic baseline generation, Scenario analysis, Sub-national detail, Land use detail, Land cover detail

## Abstract

This dataset provides projections of land use and land cover from 2020 to 2050, spanning an uncertainty range consistent with the five Shared Socio-Economic Pathways. For Europe, results are reported at both the national and the sub-national level of administrative NUTS2 regions, while regions outside Europe are represented at the national or aggregated level. The dataset distinguishes eight land cover types (e.g. cropland, pastureland, shrubland) and 65 corresponding land use categories (e.g. individual crops) and is available at two- and five-year intervals. The projections are generated using G-RDEM, a recursive-dynamic global Computable General Equilibrium model designed to capture long-term structural change on both the supply and demand sides. Implemented via the modelling platform CGEBox, G-RDEM simulates economic and land use dynamics directly at the NUTS2 level, eliminating the need for multi-model coupling and post-model downscaling used in existing SSP-based LULC quantifications. The modelling framework is open-source, reproducible, and transparent, and enables researchers to build upon or extend the projections. By outlining land transitions across socio-economic scenarios, the dataset supports research requiring detailed LULC projections as input, for example in climate impact or biodiversity conservation studies. The projections are particularly valuable for researchers and policymakers working on economically grounded assessments of long-term land system change in Europe. As an independent, scenario-based projection set grounded in a distinct economic approach, the dataset contributes to uncertainty analysis in land use and land cover futures, supports multi-model comparisons and ensemble modelling, and broadens the understanding of regional land use and land cover developments.

Specifications Table

**Table:** 

Subject	Earth & Environmental Sciences
Specific subject area	NUTS2 level projections of European land use and land cover under socio-economic scenarios for environmental assessments.
Type of data	Tabular time-series data by region, land use and land cover, and each of the five Shared Socio-Economic Pathways.
Data collection	Projections are generated using G-RDEM, a recursive-dynamic global Computable General Equilibrium (CGE) model implemented in the open-source modelling platform CGEBox. Economic and land use dynamics are simulated at the NUTS2 level under the five Shared Socio-Economic Pathways from 2020 to 2050, covering eight land cover types and 65 corresponding land use categories, reported at two- and five-year intervals.
Data source location	Geographic extent: Europe at NUTS2 and national levels; rest of the world at national or aggregated level.Main input data locations for 2017 as the benchmark point: • CGE input data: ○ https://www.gtap.agecon.purdue.edu/databases/default.asp • Land use and land cover disaggregation: ○ https://www.cia.gov/the-world-factbook/field/area/country-comparison ○ https://www.fao.org/faostat/en/#data/LC ○ https://land.copernicus.eu/en/products/corine-land-cover ○ https://www.fao.org/faostat/en/#data/FBS • NUTS2 level disaggregation: ○ https://ec.europa.eu/eurostat/databrowser/view/nama_10r_3gva/default/table?lang=en ○ https://ec.europa.eu/eurostat/databrowser/view/AGR_R_ACCTS/default/table?lang=en ○ https://ec.europa.eu/eurostat/databrowser/view/SBS_R_NUTS06_R2/default/table?lang=en ○ https://ec.europa.eu/eurostat/databrowser/view/lfst_r_lfe2en2/default/table?lang=en ○ https://ec.europa.eu/eurostat/databrowser/view/ef_lus_allcrops/default/table?lang=en ○ https://ec.europa.eu/eurostat/databrowser/view/PROJ_19RP3/default/table?lang=en ○ https://ec.europa.eu/eurostat/databrowser/view/ef_mp_irri__custom_11932000/default/table
Data accessibility	Repository name: NUTS2 Level Land Use and Land Cover Quantifications under the Shared Socio-Economic Pathways, 2020–2050 Data identification number: 10.17632/x4xfh2kcdy.1 Direct URL to data: https://data.mendeley.com/datasets/x4xfh2kcdy/1
Related research article	Goebel, T., Britz, W., 2025. Global land use and land cover projections under the shared socio-economic pathways: An integrated computable general equilibrium analysis with sub-national resolution for Europe. Glob. Environ. Change Adv., 5, 1000127. DOI: 10.1016/j.gecadv.2025.100027. [1]

## 1 Value of the Data

• The dataset provides land use and land cover (LULC) projections for European (NUTS2 and national level) and non-European (national or aggregated level) regions under the five Shared Socio-Economic Pathways (SSPs) from 2020 to 2050.
• The projections distinguish eight land cover types (e.g. cropland, pastureland, shrubland) and 65 corresponding land uses (e.g. individual crops), reported at two- and five-year intervals.
• The projections are generated with G-RDEM, a recursive-dynamic global Computable General Equilibrium (CGE) model implemented via the modelling platform CGEBox.
• The model set-up eliminates the need for multi-model coupling and post-model downscaling used in existing SSP-based LULC quantifications.
• The modelling framework is open-source, reproducible, and transparent, and enables researchers to build upon or extend the projections.
• The dataset supports research requiring detailed LULC projections as input; enables economically grounded assessments of long-term land system change in Europe; and contributes to uncertainty analysis, multi-model comparisons, and ensemble modelling of LULC futures.

## 2 Background

**Fig. 1:**
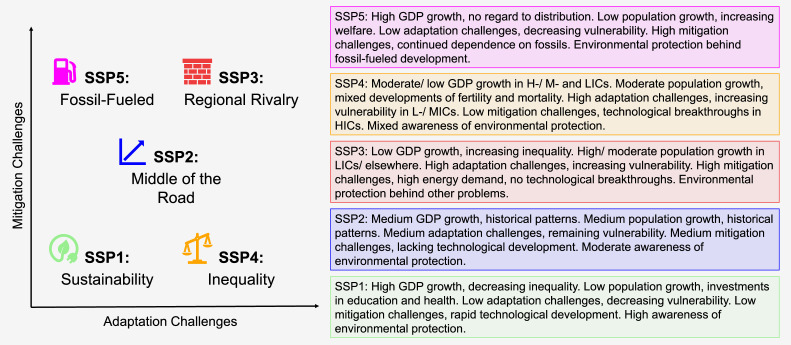
Overview of the shared socio-economic pathways.

Source: [1].

To address uncertainty in LULC developments, the climate research community explores contrasting futures by varying socio-economic trends according to the predefined narratives of the SSPs, which are graphically summarised in Fig. 1. Existing SSP-based projections derived from Integrated Assessment Models lack sufficient detail for regional and sectoral analyses. To bridge this gap, the present dataset provides LULC quantifications for the five SSPs from 2020 to 2050, offering high sectoral detail globally and sub-national resolution for Europe. The projections are generated using an open-source, recursive-dynamic global CGE model, which builds on highly disaggregated input data and includes dedicated modules for land use and energy accounting. Tailored for long-term analysis, the model explicitly considers key drivers of structural change and integrates SSP-specific projections on gross domestic product (GDP), demography, cropland, and yields. In addition, the projections reflect quantified assumptions on LULC drivers consistent with the SSP narratives.

## 3 Data Description

**Fig. 2:**
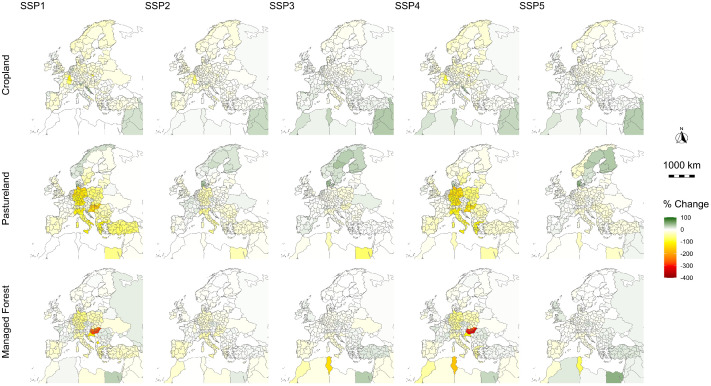
Projected changes in managed land cover under the five shared socio-economic pathways from 2020 to 2050.

Source: Own illustration. Remark: The maps are projected in the coordinate reference system WGS 84 (EPSG:4326).

The dataset follows the methodology described in Goebel and Britz [1] and provides LULC projections at the level of administrative NUTS2 regions for the five SSPs [2] for the period 2020 to 2050. European Union (EU) member states follow the 2016 NUTS2 classification [3], while European Free Trade Association (EFTA) countries and EU accession candidates follow the 2008 classification [4]. Overseas territories are excluded. In total, the dataset covers 317 NUTS2 regions for Europe, which are listed and mapped in the accompanying data explanation document. In addition to the sub-national projections, the dataset includes matching national level results and covers the rest of the world at national or aggregated level. The projected LULC categories are based on version 11 of the Global Trade Analysis Project (GTAP) dataset [5], with further disaggregation as detailed in [1]. Table 1 lists the LULC categories included, while Fig. 2 illustrates the projected changes in managed land covers across Europe. The dataset is available via Mendeley Data [6] in both .csv and a .xlsx formats, with two- and five-year resolution. It is accompanied by a data explanation document, also available in .csv and .xlsx formats.

**Table 1:** Land use and land cover detail in the dataset.

Land Cover	Managed	Land Use			
Cropland	Yes	- Apples - Bananas/ plantains - Barley - Citrus fruits - Cocoa beans - Coffee beans - Grapes - Leguminosae - Maize	- Nuts - Oats - Olives - Other cereals - Other crops - Other fruits - Other oilseeds - Other roots/ tubers - Other vegetables	- Paddy rice - Palm oil fruit - Plant-based fibres - Potatoes - Rape seed - Rye - Sorghum - Soy bean - Sugar cane/ beet	- Teas - Tomatoes - Wheat All as irrigated and rainfed variants.
Pastureland	Yes	- Cattle for meat	- Ruminants for meat	- Raw milk	- Wool
Managed forest	Yes	- Forestry			
Natural forest	No				
Savannah and grassland	No				
Shrubland	No				
Other land	No				
Built-up land	No				

Source: [1].

## 4 Experimental Design, Materials and Methods

This dataset provides LULC projections at the level of administrative NUTS2 regions for the five SSPs from 2020 to 2050. Each projection is part of a recursive-dynamic CGE baseline generated with G-RDEM [7], the GTAP-based recursive-dynamic economic model. Global CGE models represent the entire economy, including international trade, and are therefore well-suited to analyse the impact of interrelated socio-economic drivers on LULC dynamics. However, they typically operate at coarse regional and sectoral resolution. This limitation is addressed in this study by operating G-RDEM via the open-source modelling platform CGEBox [8,9].^1^ This set-up enables the simulation of economic and land use dynamics directly at the NUTS2 level and allows the integration of model extensions which enhance LULC representation and enable energy accounting. The CGE baselines underlying the projections differ according to the SSP narratives, which describe contrasting socio-economic developments associated with different challenges to climate change adaptation and mitigation. They are incorporated as follows:

(1) **Macro-economic developments** (GDP and demographics) follow country- and scenario-specific projections from version 3.1 of the OECD Env-Growth model and the WiC-POP model [10].
(2) **LULC drivers** are parameterised using an SSP-specific assumption catalogue consistent with the SSP narratives [1].

The benchmark dataset underlying the CGE simulations builds on the GTAP v11 Power database [5], which provides a snapshot of the global economy in 2017. For this study, the full sectoral detail of this database is maintained, while electricity products and labour-related production factors are aggregated. Land use is further disaggregated to the detail shown in Table 1, and irrigation water is introduced as an additional production factor to distinguish between irrigated and rainfed cropland production. Outside this study’s focus region,^2^ countries are aggregated into larger model regions to facilitate comparison with existing global projections. Within the European focus region, production and factor use, including land use, are disaggregated to the level of administrative NUTS2 regions. A detailed description of the disaggregated CGE input data, the model set-up, and the SSP implementation is provided in Goebel and Britz [1].^3^ This article focuses on the processing of LULC and NUTS2 level data for the use in long-term baseline generation and publishes the resulting NUTS2 level LULC projections.

### 4.1 Land use and land cover representation

To improve the representation of LULC, the simulations use CGEBox’ Agro-Ecological Zone (AEZ) module, which builds on the GTAP-AEZ model [12], but extends it in three ways [9]:

(1) **Land supply** is modelled by a nested multi-level Constant Elasticity of Transformation (CET) function. At the top level, an additive functional form is used to preserve physical area balances.^4^
(2) **Cropland nesting** includes an additional layer to differentiate transformations between annual and perennial crops.
(3) **Cropland conversion** from pastureland, managed forest, or unmanaged land is determined based on a statistically estimated correlation in historical regional land conversion data.

The GTAP v11 AEZ dataset [13] provides the base LULC input data. It links physical LULC to the GTAP v11 Power database by dividing land into 18 AEZs with similar cultivation conditions and distinguishes seven land cover types and 13 land uses.^5^ Our work systematically improves the LULC input during database generation in CGEBox to produce consistent CGE-based LULC projections.^6^ A set of a-priori target values is constructed for four key corrections:

(1) **Total area adjustment:** GTAP v11 AEZ total areas are compared with World Factbook statistics [14]. Country level areas are replaced by official statistics, and AEZ level areas are recalculated based on GTAP v11 AEZ shares.^7^
(2) **Unmanaged forest land:** The GTAP v11 AEZ data underestimates forest area, as it only includes managed forest within ten kilometres of infrastructure [13]. The difference between GTAP v11 AEZ reported total land areas and official statistics is filled by introducing an “unmanaged forest” land cover category, informed by FAOSTAT tree cover statistics [15]. Remaining total area discrepancies are assigned to the land cover type “other land”.
(3) **European land cover refinement:** Detailed 2018 CORINE land cover data [16] are aggregated to country and AEZ level using GIS analysis in R, replacing the GTAP v11 AEZ data for Europe.^8^
(4) **Data inconsistencies:** Several internal inconsistencies in the GTAP v11 AEZ data are corrected, including removal of economic returns without corresponding land cover, estimation of missing land use entries using proportionality assumptions, and deletion of unmatched records.

The corrected GTAP v11 AEZ data is rebalanced by solving a non-linear programming (NLP) problem which minimises the squared relative differences to the a-priori target values using a highest posterior density (HPD) objective function. The rebalanced dataset satisfies six conditions:

(1) Posterior land rents for each sector aggregated over the AEZs equal the sum of land rents reported in the GTAP v11 Power database.
(2) Posterior land rents per hectare, multiplied by areas, equal posterior activity level land rents.
(3) Posterior managed land area equals the sum of all posterior land use activity areas.
(4) Posterior total area equals the sum of all related posterior land covers.
(5) Posterior total forest area equals posterior managed plus posterior unmanaged forest area.
(6) Each region includes at least one percent of posterior unmanaged land to avoid numerical issues.

After correction of the input data, two data disaggregation steps are applied to enhance LULC detail:

(1) **Agro-food split**: Following Britz [17],^9^ the eight GTAP v11 cropland production activities and sectors are disaggregated into 30, and the four pastureland production activities and sectors into eight. A detailed mapping between GTAP v11 and the disaggregated entries is provided in the data explanation document provided in the data repository.
(2) **Irrigation split:** The production factor land is split to introduce irrigation water as a new production factor and the 30 cropland production activities are disaggregated into irrigated and rainfed variants using FAO irrigation statistics [19] and a methodology similar to the one used in the agro-food split.

The final database distinguishes eight land covers and 65 land uses (Table 1), corresponding to the LULC level of detail in the dataset presented here.

### 4.2 NUTS2 representation

Harmonised economic activity data at sub-national level reported by EUROSTAT are limited, and not available for demand and bilateral trade. To address this, CGEBox includes a module which integrates sub-national detail where data is available, while solving the remaining model domains at the national level. Specifically, production functions and commodity output are represented at the sub-national (NUTS2) level, while market clearing including commodity demand is represented at the national level. To satisfy equilibrium conditions and reflect quality differences, sub-national production volumes are aggregated to the national level using a Constant Elasticity of Substitution (CES) aggregator. Factor markets are represented at sub-national level: land and water are region specific, while capital and labour are sluggishly mobile across regions. Accordingly, factor availability, mobility, and prices differ sub-nationally based on CET factor supply functions, while national intermediate input prices apply uniformly across sub-regions, as sub-national trade margins are not considered. Taxes are modelled exclusively at the national level, and therefore apply uniformly across all sub-regions. As a result, the sub-national data requirements are relatively modest, consisting essentially of output quantities and cost shares net of taxes. Prior to database generation in CGEBox, three categories of sub-national data are collected and prepared^10^:

(1) **Gross value added (GVA) data for all GTAP sectors at NUTS2 level**: Since national accounts do not provide sectoral production values at sub-national level, this work relies on NUTS2 GVA data together with the assumption of equal sectoral cost structures across regions. EUROSTAT provides GVA at NUTS3 level for eleven broad sectors [20], which are aggregated to NUTS2 level. These broad sector data are then disaggregated to GTAP sector detail using additional data sources:
  • *Agriculture (part of sector A):* NUTS2 level production shares are taken from the Economic Accounts for Agriculture (EAA) [21]. Where unavailable, disaggregation is based on NUTS2 level land cover shares derived from a GIS intersection of CORINE 2018 land cover data [16] with 2016 NUTS2 boundaries [22].
  • *Manufacturing (sector C), energy/ utilities/ waste (sector BDE), construction (sector F), retail/ logistics/ hospitality (sector GI), information and communication (sector J), finance and insurance (sector K), real estate (sector L), business services (sector MN), and community/ social/ personal services (sector RU):* disaggregation is based on NUTS2 level salary shares from EUROSTAT [23].
  • *Public services (sector OQ):* disaggregation uses NUTS2 level employment shares [24].

**Fig. 3:**
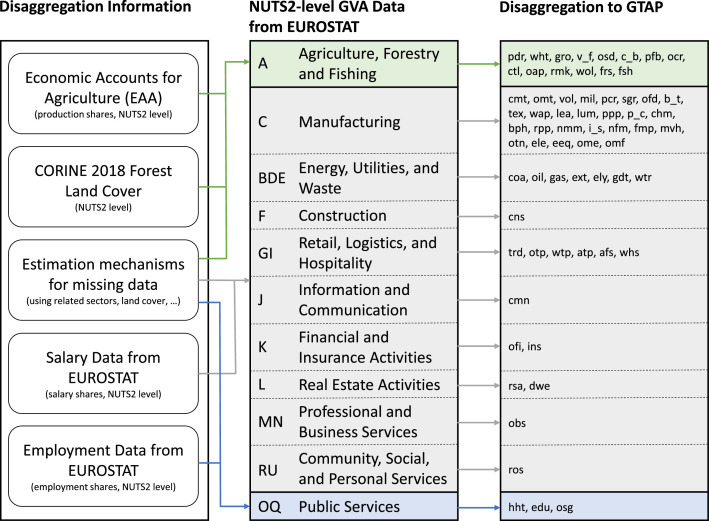
Processing of NUTS2 gross value added data.

Source: Own illustration.

Where no NUTS2 level data is available, disaggregation of the broad GVA data to GTAP sectoral level is inferred from related sectors or GVA records are distributed evenly across GTAP sectors. Fig. 3 illustrates the GVA preparation process.

(1) **LULC data at NUTS2 level:** Production factor stocks are disaggregated to NUTS2 level using the equal cost structure assumption: national stocks by sector are allocated according to NUTS2 production shares. For land, this procedure is refined by overwriting factor stocks with GIS-derived land cover information from CORINE 2018 land cover data [16] intersected with the 2016 NUTS2 boundaries [22]. Where available, NUTS2 level Farm Structure Survey (FSS) data [25] are used to further refine cropland and pastureland entries and to provide land use information at NUTS2 level.
(2) **Additional data for optional use:** Population projections at NUTS2 level [26] are provided, which can be used to disaggregate SSP-specific population projections to sub-national level during baseline generation. Further, NUTS2 level irrigation areas [27] are included for applications involving water splits, and NUTS2 level AEZ shares are provided based on GIS analysis.

The processed NUTS2 data are stored in a GAMS Data Exchange (GDX)^11^ file and are automatically imported when the CGEBox framework generates a database which contains European countries as single entities.^12^ The model first adjusts the NUTS2 GVA data to the current aggregation and performs consistency checks. For example, NUTS2 data entries are deleted if no corresponding national production exists in the GTAP v11 database, and very small data entries are removed to avoid numerical issues. As at the national level, NUTS2 LULC data are rebalanced by solving a NLP problem minimising the squared relative differences to the a priori target values, equivalent of maximizing the posterior density under normally distributed relative errors. The rebalanced data meet the following conditions:

(1) Posterior AEZ and NUTS2 level land rents aggregated over AEZs equal NUTS2 level land rents.
(2) Posterior NUTS2 level land rents aggregated over NUTS2 equal national land rents.
(3) Posterior NUTS2 level managed land area aggregated over NUTS2 equals national managed land areas.
(4) Posterior NUTS2 level unmanaged land area aggregated over NUTS2 equals national unmanaged land areas.
(5) Posterior total area equals the sum of all related posterior land covers.
(6) Posterior areas aggregated over AEZs, NUTS2, and land cover equal national total land areas.

The production side of the LULC-modified GTAP data is disaggregated to sub-national level by applying NUTS2 shares to national entries. The final input dataset comprises 47 countries or regions, 317 NUTS2 regions, 143 production activities, 103 products, and six production factors, forming the basis for the projections presented here.

## Limitations

The dataset provides detailed projections of LULC under the five SSPs from 2020 to 2050. These projections depend on exogenous projections, assumptions on SSP-specific LULC drivers, the CGE input data, and the model’s mechanisms and parameterisation. Each of these elements carries uncertainties, though projections remain internally consistent according to CGE balancing and accounting conditions. The SSPs illustrate contrasting futures without implying that these five narratives are the only plausible ones or attaching probabilities to them. Our scenario-based projections therefore reflect potential LULC developments rather than exact predictions. Sub-national data follow the 2016 NUTS2 classification, consistent with the 2017 base year of the GTAP v11 database. However, users should note that more recent NUTS2 classifications (2021 and 2024) differ slightly and that overseas territories are excluded from the projections. The equal cost structure assumption across sub-national units and uncertainties in secondary data sources (e.g. CORINE 2018 land cover) may reduce precision at sub-national level. Model parameters - particularly elasticities in CES and CET structures - are uncertain and influence projected LULC dynamics. Outside Europe, regions are represented at national or aggregated levels, which masks local heterogeneity. Users should consider these factors when applying the dataset for research, modelling, or policy support.

## Ethics Statement

The authors have read and follow the ethical requirements for publication in Data in Brief. The current work does not involve human subjects, animal experiments, or any data collected from social media platforms.

## CRediT Author Statement

**Theresa Goebel:** Writing - original draft, Visualization, Software, Methodology, Formal analysis, Conceptualisation. **Wolfgang Britz:** Writing - review and editing, Supervision, Software, Methodology, Conceptualisation.

## Acknowledgements

This work was supported by the Deutsche Forschungsgemeinschaft (DFG, German Research Foundation) under project SFB 1502/1–2022 – project number 450,058,266 (DETECT).

## Data Availability

Mendeley DataNUTS2 Level Land Use and Land Cover Quantifications under the Shared Socio-Economic Pathways, 2020-2050 (Original data).

## Footnotes

4 At the lower levels, projections are scaled retrospectively to add up to the top levels.

7 Data wrangling is conducted in R. Target values are provided to the model via CGEBox\data\aezArea_v11.gdx.

8 The mapping between CORINE and GTAP land cover classes is shown in the data explanation document. Target values are provided to the model via CGEBox\data\CORINE_AEZ.gdx.

9 Based on more recent input data from [18].

## Footnotes

1 The model documentation provides necessary information for downloading and using CGEBox. The related research article [1] adds information on the model version used for the LULC quantifications, on the database generation process, and on the model setup. In the following, reference is made to relevant code sections referring to the respective directory in CGEBox.

2 The study’s focus region is the EURO—CORDEX domain [11].

3 Reference [1] (in particular Appendix B) provides a detailed description of how to replicate this work.

5 GTAP v11 AEZ land cover comprises cropland, pastureland, forest, savannah and grassland, shrubland, other land (rock, ice, desert) and built-up land. GTAP v11 AEZ land use comprises eight cropland, four pastureland and one forest production activity/ sector. For more detail see the data explanation document.

6 The code correcting GTAP v11 AEZ data can be found in CGEBox\gams\gtapAgg\aggAez.gms.

10 The code preparing NUTS2 level data can be found in CGEBox\gams\GTAPNuts2\createNUTS2_V11.gms.

11 CGEBox\gams\GTAPNuts2\gams\GTAPNuts2\gdp_NUTS2_V11.gdx

12 For numerical reasons, the disaggregation of a database at NUTS2 level is only carried out in the final step of multi-step data processing procedures.
